# Welfare effect analysis of pay-as-you-go pension system: Deconstruction from the perspective of relative utility and social equality

**DOI:** 10.1371/journal.pone.0296334

**Published:** 2024-05-10

**Authors:** Jin Hu, Peter J. Stauvermann

**Affiliations:** 1 Dept. of Economics, Jiaxing University, Jiaxing, Zhejiang Province, China; 2 Dept. of Global Business & Economics, Changwon National University, Changwon, Republic of Korea; UCL: University College London, UNITED KINGDOM

## Abstract

This paper studies the redistributive effects of two major pay-as-you-go pension systems by constructing an intergenerational iterative model which does not only considers standard utility but also relative utility. The study find that the two main pay-as-you-go pension systems are both sustainable. If we consider different preferences, then the choice of pension system should depend on the question of whether individuals are more interested in the absolute level of consumption or in the consumption related to a reference group. If the latter is more important, the Beveridgean system is superior, it provides greater protection for vulnerable groups than the Bismarck pension system, and the pension income after retirement is relatively more balanced, but the price is a lower level of consumption in the long run compared to an economy with Bismarckian system. If individuals prefer instead the absolute level of consumption, the Bismarckian system is better, because it guarantees a comparable higher level of consumption, but the disadvantaged groups face a higher risk of poverty and the degree of social inequality will be relatively higher. However, it is important to note that in the long run, only the level of consumption differs, not the speed of growth or number of children.

## 1. Introduction

In countries that operate under a pay as you go pension (PAYG) system, the question of its sustainability stands out as one of the most crucial issues. The concern arises from the fear that the pension system may collapse due to an unsustainable fertility rate and increasing life expectancy.

The basis of this consideration lies in the mechanics of the PAYG system, which operates by using the current contributions of employees to pay pension benefits to the current retired generation. As life expectancy increases, the number of retired individuals also rises, given a constant retirement age. Simultaneously, a decline in the number of children leads to a reduction in contributors to the pension system. Consequently, there is an imbalance between revenue and expenditure, where expenditures increase while revenue declines, all other factors remaining constant.

The usual line of argument, as presented by Stauvermann and Kumar [1], revolves around the impact of the decreasing ratio between workers and retired individuals in a pay-as-you-go pension system. They assert that if this ratio declines, there are two potential outcomes: either the contribution rate must increase, which could impose economic burdens on the working generation, or the pensions will gradually decline over time, leading to severe consequences such as poverty and malnourishment for the elderly.

Sinn highlights an extreme example of this oversimplified view concerning OECD countries [2]. He predicts that these countries will witness a doubling in the number of elderly people relative to the young within the next thirty years. Consequently, the implication for the pay-as-you-go system appears straightforward. Either the contribution rate must be doubled to maintain pensions in line with wages, or the pensions will need to be halved in relation to wages.”

According to Stauvermann and Kumar, the arguments put forth by Sinn suffer from inconsistency. Initially, Sinn presents a standard neoclassical perspective, advocating for the necessary increase in the contribution rate. However, he later shifts his viewpoint to that of the relative utility (Duesenberry 1949, Hollander 2000, Ferrer-i-Carbonell 2005, Luttmer 2005, Frank 2007, Clark 2008, Gruber 2018 [3–9]) by referring to the pension-to-wage ratio. It’s worth noting that Sinn is not the only one who adopts this inconsistent approach, as even the OECD supports the idea that the pension-to-wage ratio holds significant relevance in the pension debate. This is surprising, given that our hypothesis, derived from Hollander, suggests that in a status-oriented world, the extent of the contribution rate is inconsequential as long as all agents face the same rates.

However, for a neoclassical economist, the pension-to-wage ratio becomes utterly irrelevant since what matters is solely the absolute purchasing power of the pension payout concerning individual utility.

Schoeck [10] and Rawls [11] highlight the importance of status-seeking as a significant characteristic of human behavior. The notion that the overall level of satisfaction or subjective well-being derived from a certain level of consumption depends not only on the consumption level itself but also on how it compares to the consumption of other members of society is not a new concept in economics. While its origins can be traced as far back as Marx [12], Smith [13], and Veblen [14], it was not until the work of Duesenberry [3] and Pollak [15] that an attempt was made to provide this idea with some microeconomic foundations.

The primary objective of this paper is to explore the theoretical aspects and provide an illustration regarding the sustainability of the pay-as-you-go (PAYG) pension system. In doing so, we will consider both the standard utility approach and the relative utility approach, with the latter tracing its roots back to Duesenberry (1949) and gaining significant attention through the work of Easterlin [16, 17].

Furthermore, our investigation goes beyond the scope of Stauvermann and Kumar [1] by not only considering the Bismarckian PAYG pension system but also taking into account the Beveridgean PAYG pension system. The key distinction between these two systems lies in the presence of an intra-generational redistributive element in the Beveridgean scheme, which is absent in the Bismarckian system.

In the Beveridgean scheme, contributions are proportional to income, while the pension benefit is identical for all retired individuals. As a result, high-income individuals contribute more than their less affluent counterparts but receive the same pension benefit. Conversely, in the Bismarckian scheme, both individual contributions and pension benefits are proportional. This leads to high-income individuals receiving a proportionally higher pension benefit than those with lower incomes.

It is important to acknowledge that the apparent redistribution of contributions from the rich to the poor in the Beveridgean system and the perceived lack of redistribution in the Bismarckian system are subject to a crucial condition: the assumption that life expectancy is the same for both rich and poor individuals.

However, recent research by Stauvermann et al. (2023) [18] reveal that the Bismarckian system, in fact, exhibits a regressive impact. This occurs because in many developed countries, high-income individuals tend to have significantly higher life expectancies than low-income individuals. For instance, in Germany, the difference in life expectancies is roughly 10 years. Consequently, due to their lower life expectancy, the aggregated benefits received by the poor are disproportionately low, while those received by the rich are disproportionately high. This leads to a redistribution of contributions from low-income individuals to high-income individuals within the Bismarckian system.

Similarly, the redistributive effect of the Beveridgean system is also impacted, at the very least, by the different life expectancies of poor and rich individuals. If the difference in life expectancies increases, it is plausible that the Beveridgean system may also become regressive.

In summary, the relationship between pension systems, life expectancy, and redistributive effects is complex and underscores the need to consider these factors when evaluating the true impact of different pension schemes.

In contrast to Stauvermann and Kumar [1], our analysis takes into account a heterogeneous population with varying incomes or productivity, recognizing the crucial role played by these diverse individuals in the overall analysis. Additionally, we expand the scope of our investigation by incorporating the Beveridgean pension system.

The consideration of a heterogeneous population allows us to account for the different income levels and productivity of individuals, providing a more comprehensive understanding of the implications of the pension system on various segments of society.

Moreover, the inclusion of the Beveridgean pension system further enriches our analysis, as it introduces a different set of dynamics and redistributive elements compared to the Bismarckian system. This extension broadens the scope of our research, enabling us to explore the impacts of different pension models on social welfare and equality within the heterogeneous population.

By considering both the heterogeneity of the population and the two distinct pension systems, our study aims to offer a more nuanced and comprehensive assessment of the sustainability and redistributive effects of the PAYG pension system.

In a society characterized by heterogeneous individuals, it becomes essential to incorporate not only the standard utility approach but also the relative utility approach when considering their subjective well-being. This is because people’s happiness and satisfaction are influenced not only by their absolute income within society but also by their relative income or social status in comparison to others and the past.

Hollander [4] extensively examines the validity of utility statements using these two approaches. He concludes that standard utility theory is either unrelated to individual experiences, and thus must be asserted as valid a priori, or it becomes highly problematic when tested empirically. In contrast, Duesenberry’s status-oriented utility theory finds support from available data on subjective well-being, making it a more viable and applicable framework to understand the dynamics of human happiness and satisfaction within a society of diverse individuals.

The remainder of the paper is structured as follows: In the subsequent section, we provide a brief review of the pertinent literature. Subsequently, we introduce the model and outline the economic consequences of the two pension schemes under both the standard utility approach and the relative utility approach. Finally, we present our conclusions in the last section.

## 2. Literature review

### 2.1 Pay as you go pension system, equality between generations and equality within generations

The pay-as-you-go pension system operates on the premise that the pension revenue for retirees comes from the pension contributions made by the current working generation. This transfer mechanism facilitates the extension of consumption from youth to old age. Samuelson’s intergenerational general equilibrium model [19] was one of the earliest classical works to explore the redistribution function of the pay-as-you-go pension system, addressing income transfers during individuals’ working and retirement periods and between different generations.

Hindriks & De Donder [20] emphasize that the pay-as-you-go pension system holds a crucial role in modern social security due to its intergenerational redistribution effects on both the young and the elderly. Furthermore, Zhang et al. [21], using the intergenerational iterative model, demonstrated that the pay-as-you-go pension system can remain stable even with a decreasing population and increasing human capital.

Addressing pension reform, Meyer & Bridgen [22] highlight the importance of tackling poverty among the elderly and addressing inequality within the pension generation. They argue that a basic pension system, with pay-as-you-go as its main component, is a fundamental and universal institutional arrangement. The absence of such a system could lead to a significant rise in the elderly poverty rate.

For instance, Zheng [23] indicates that in the United States, the basic pension system helps keep the elderly poverty rate at 11%, but without it, the rate could soar to 60%. Similarly, in China, the elderly poverty rate would exceed 90% without a basic pension system. Therefore, the pay-as-you-go pension system plays a vital role in ensuring social security.

Most countries have implemented a pay-as-you-go (PAYG) pension system due to its immediate advantage of being able to provide pension benefits soon after the system’s inception. In contrast, a capital-funded pension system necessitates years of savings accumulation before benefits can be disbursed. While the PAYG system offers this advantage, it also comes with the disadvantage that transitioning from a PAYG system to a capital-funded one becomes nearly impossible. Such a shift would require at least one young generation to build a substantial fund while simultaneously financing the pension benefits of the older generation, who contributed to the PAYG pension system.

A capital-funded pension system can be organized either privately or by the government. However, in both cases, the extent of pension benefits depends on the development of financial markets, introducing a risk for participants in the pension system.

Furthermore, capital-funded pension benefits are contingent on income earned during the working years, leading to greater retirement savings and investment revenue for individuals with higher salaries. Consequently, this exacerbates intra-generational inequality as those with higher incomes tend to accumulate more substantial pension savings.

### 2.2 Pay as you go pension system, individual differences and social equality

To understand the impact of the pension system on income equality, it is crucial to consider the influence of individual heterogeneity. Numerous studies have indicated that the pension system tends to widen the income gap, as individuals with more wealth, human capital, and better health tend to accumulate greater wealth compared to those with little or no wealth, limited human capital, and poorer health conditions.

Using savings panel data from Germany, Pfarr & Schneider [24] observed that the introduction of government incentives, such as subsidies and tax credits, resulted in varying private pension development among different groups. Individuals with more assets and higher incomes were more willing to invest in private pensions. However, for those with limited assets and low incomes, the impact of subsidies on personal pension savings was not significant.

Moreover, Pestieau & Ponthiere [25] investigated the equality of the pension system from the perspective of life expectancy. They concluded that individuals with higher incomes tend to live longer than those with lower incomes. As a result, the funded pension system and private pension schemes exacerbate income inequality.

Considering these findings, a pay-as-you-go basic pension system that accounts for the needs of disadvantaged groups becomes crucial to ensuring social equity among heterogeneous individuals. By providing support to those with fewer resources, this type of pension system can help mitigate income disparities and promote a more equitable society.

When the basic pension covers only a limited proportion of the population, vulnerable groups may be excluded from the public pension system, leading to a widening income gap. In this case, income inequality will increase further if the middle class and the wealthy join the private pension system.

### 2.3 Pay-as-you-go pension system and relative utility perspective

The current research on pay-as-you-go pension systems primarily focuses on standard utility, with only a few considering the perspective of relative utility. However, understanding the relativity of utility is crucial as it plays a significant role in human behavior. The overall satisfaction or subjective well-being derived from a particular level of consumption depends not only on the consumption level itself but also on how it compares with others in society.

Duesenberry [3] introduced the relative income hypothesis in his PhD dissertation and proposed a consumption function that takes into account the current income of other people. According to his argument, ’for any given relative income distribution, the percentage of income saved by a family will tend to be unique, invariant, and an increasing function of its percentile position in the income distribution. The percentage saved will be independent of the absolute level of income. It follows that the aggregate saving ratio will be independent of the absolute level of income’.

Another significant research driving the development of the relative income theory is the Easterlin paradox [16, 17]. Richard Easterlin found that despite a dramatic rise in the income of most European countries after World War II, their happiness levels did not experience a proportional increase. Although, within a country at a given point in time, those with higher incomes are, on average, happier than those with lower incomes. However, the average happiness in developed countries has remained relatively constant over time, despite sharp increases in per capita GDP. Clark et al. [8] emphasize the importance of interpersonal comparisons to account for ’Easterlin’ s paradox.’

Several other researchers have also estimated the direct impact of interpersonal comparisons on subjective well-being. For instance, Luttmer [6], by analyzing individual-level panel data on well-being from the U.S. National Survey of Families and Households, concludes that, after controlling for income and other individual characteristics, the local average earnings have a significantly negative effect on subjective happiness. Using data from a large German panel data set, the German Socio-Economic Panel (GSOEP), Ferrer-i-Carbonell [5] finds that the income of the reference group is about as important as one’s own income for subjective well-being. Similarly, Dynan and Ravina [26] report similar results for US households, indicating that people’s subjective well-being depends positively on how well they are doing relative to others around them, after controlling for their own income levels. In a related work, Frey and Stutzer [27] argue that when absolute income reaches a certain level, relative income becomes much more critical for subjective well-being than absolute income itself.

Cheng [28] conducted a detailed analysis of the relationship between the pay-as-you-go system and population aging from the perspective of relative utility. They established a theoretical model of the pension system, demonstrating that population aging does not necessarily lead to a payment crisis in the pay-as-you-go system. Instead, the crucial factor lies in the relative gap between the speed of economic growth and the speed of population aging.

Furthermore, the standard utility approach and the relative utility approach often yield diametrically opposed welfare outcomes. For instance, Duesenberry’s relative utility hypothesis suggests that a pay-as-you-go pension system might enhance economic efficiency, contrary to what the standard utility approach implies. This highlights the significance of incorporating relative utility into the utility function, offering a fresh perspective for analyzing various economic behaviors, particularly the growing income inequality.

Thus, our paper aims to delve into the economic implications of two distinct pension systems while taking into account both the standard utility approach and the relative utility approach. In other words, we seek to analyze how individuals perceive and value the two types of pension systems within the framework of these two approaches.

## 3. The model

We begin with human capital building of an individual *i* by assuming the following human capital function:

$$h_{t+1}^{i}=Bh_{t}^{i}{\left(q_{t}^{i}\right)}^{\varepsilon },\;\forall i.$$ (3.1)

Still the human capital of an individual depends on the human capital of the parents and the time devoted for education expressed in terms of income of parents. To keep the analysis as simple as possible, we assume that the society consists only of two types of individuals, individuals of low and high ability, where the superscripts *l* and *h* identify them. Thus, we assume:

$$h_{t}^{h}>h_{t}^{l},\;\forall i.$$ (3.2)

In all other respects regarding preferences the individuals are assumed being identical. Further we assume, that *ψ*_*t*_ ∈ [0, 1] is the share of low ability individuals in the population and 1 − *ψ*_*t*_ is the share of high ability individuals in the population. Every individual born in *t*-1 maximizes the following utility function:

$$\begin{array}{l} U_{t}\left(c_{t}^{i1},c_{t+1}^{i2},n_{t},q_{t}^{i}\right)=\eta \;ln\left(c_{t}^{i1}\right)+\left(1-\eta \right)ln\left(\frac{c_{t}^{i1}}{{\bar{c}}_{t}}\right)+\\ \chi (\eta \;ln\left(c_{t+1}^{i2}\right)+\left(1-\eta \right)ln\left(\frac{c_{t+1}^{i2}}{{\bar{c}}_{t+1}}\right)+\phi \left(ln\left(n_{t}^{i}\right)+\nu ln\left(h_{t+1}^{i}\right)\right) \end{array}$$ (3.3)

This utility function differs in some respect from the ones, introduced earlier. The parameter *η* ∈ [0, 1] indicates the weight of the importance of the absolute consumption, while 1 − *η* indicates the weight of the importance of the status consumption. If *η* = 0, only the relative consumption matters with respect to the well-being and the absolute consumption plays no role. Then again if *η* = 1 only the absolute level of consumption matters regarding the well-being. Like above $c_{t}^{i1}$ is the consumption in the second period of life, $c_{t+1}^{i2}$ is the consumption in the third period of life, $n_{t}^{i}$ is the number of children. The variable ${\bar{c}}_{t}$ is the reference consumption, which could be the average consumption of individuals who are in the second period of life, ${\bar{c}}_{t+1}$ is the reference consumption of individuals who are in the third period of life; again *χ* ∈ [0, 1] represents the subjective discount factor; the variable *ϕ* ∈ [0, 1] is the preference parameter for the quantity of children and the product ϕ*ν* ∈ [0, 1] is the preference parameter for the quality of children. The relevant budget constraints are:

$$c_{t}^{i1}=w_{t}h_{t}^{i}\left(1-\tau_{t}^{P}-\left(e+q_{t}^{i}\right)n_{t}^{i}\right)-s_{t}^{i}$$ (3.4)

$$c_{t+1}^{i2}=R_{t}s_{t}^{i}+P_{t+1}^{i}$$ (3.5)

Where *w*_*t*_ is the wage rate per human capital capita, $\tau_{t}^{P}$ is the pension contribution rate, *e* is the pure child rearing cost, $s_{t}^{i}$ is the savings an individual make in the second period of life, *R*_*t*_ is the interest factor for the savings in the second period, and $P_{t+1}^{i}$ is the pension benefit one could receive after retirement in the third period of life.

Thus, a representative agent maximizes her utility (3.3) with regard to the restrictions (3.1), (3.4) and (3.5). We insert (3.1), (3.4) and (3.5) in the utility function and solve for the following maximization problem:

$$\begin{array}{l} \;max_{\left\{s_{t}^{i},n_{t}^{i},\;q_{t}^{i}\right\}}\;U_{t}\left(s_{t}^{i}\;,q_{t}^{i},n_{t}^{i}\right)=\;\eta \;\text{ln}\left(w_{t}h_{t}^{i}\left(1-\tau_{t}^{P}-\left(e+q_{t}^{i}\right)n_{t}^{i}\right)-s_{t}^{i}\right)+\\ \left(1-\eta \right)ln\left(\frac{\left(1-\tau_{t}^{P}-\left(e+q_{t}^{i}\right)n_{t}^{i}\right)-s_{t}^{i}c_{t}^{1}}{{\bar{c}}_{t}}\right)+\chi (\eta \;\text{ln}\left(R_{t}s_{t}^{i}+P_{t+1}^{i}\right)+\\ +\left(1-\eta \right)ln\left(\frac{R_{t}s_{t}^{i}+P_{t+1}^{i}}{{\bar{c}}_{t+1}}\right))+\phi \left(ln\left(n_{t}^{i}\right)+\nu ln\left(Bh_{t}^{i}{\left(q_{t}^{i}\right)}^{\varepsilon }\right)\right) \end{array}$$ (3.6)

Differentiating with respect to the three choice variables lead to the following FOCs:

$$\frac{\chi R_{t}}{R_{t}s_{t}^{i}+P_{t+1}^{i}}=\frac{1}{w_{t}h_{t}^{i}\left(1-\tau_{t}^{P}-\left(e+q_{t}^{i}\right)n_{t}^{i}\right)-s_{t}^{i}},$$ (3.7)

$$\frac{\phi }{n_{t}^{i}}=\frac{w_{t}h_{t}^{i}\left(e+q_{t}^{i}\right)}{w_{t}h_{t}^{i}\left(1-\tau_{t}^{P}-\left(e+q_{t}^{i}\right)n_{t}^{i}\right)-s_{t}^{i}},$$ (3.8)

$$\frac{\phi \nu \epsilon }{q_{t}^{i}}=\frac{w_{t}h_{t}^{i}n_{t}^{i}}{w_{t}h_{t}^{i}\left(1-\tau_{t}^{P}-\left(e+q_{t}^{i}\right)n_{t}^{i}\right)-s_{t}^{i}}.$$ (3.9)

Solving these three equations for the choice variables deliver:

$$s_{t}^{i}=\frac{\chi w_{t}h_{t}^{i}\left(1-\tau_{t}^{P}\right)-\left(1+\phi \right)P_{t+1}^{i}}{R_{t}\left(1+\chi +\phi \right)}$$ (3.10)

$$n_{t}^{i}=\frac{\phi \left(1-\varepsilon \nu \right)\left(\left(1-\tau_{t}^{P}\right)R_{t}w_{t}h_{t}^{i}+P_{t+1}^{i}\right)}{R_{t}w_{t}h_{t}^{i}e\left(1+\chi +\phi \right)},$$ (3.11)

$$q_{t}^{i}=\frac{\varepsilon \nu e}{\left(1-\varepsilon \nu \right)}.$$ (3.12)

The optimal level of investment in education delivers the individual growth factor of education, if we insert (3.12) in (3.1);

$$G^{h}=\;\frac{h_{t+1}^{i}}{h_{t}^{i}}=B{\left(\frac{\varepsilon \nu e}{\left(1-\varepsilon \nu \right)}\right)}^{\varepsilon }.$$ (3.13)

It should be noted that this solution represents only a utility maximum, if *ενϵ*]0,1[ is satisfied. This condition is necessary and sufficient. Result (3.13) makes clear, that the growth rate of human capital is equal for all members of the economy, and is not dependent on human capital of parents.

The first important finding is:

**Proposition 1**: Regarding the optimal values of the savings, number of children and investments in human capital of children, it does not matter to which extent the individuals are status-oriented.

This outcome is in line with the considerations of Hollander (2000). That means from observing the behavior of individuals we cannot deduce if a person is status-oriented or not.

## 4. The Bismarckian pension scheme

To analyze the sustainability of the pension system we begin with Bismarckian one. We assume that the contribution rate $\tau_{t}^{P}=\tau^{P}$ is fixed and unique. Further, the pension benefits have to be proportional to the individual contributions and we assume that the system is in balance. The average pension benefit in *t*+1 $\;{\bar{P}}_{t+1}$ is given by:

$${\bar{P}}_{t+1}=\tau^{P}G^{h}w_{t+1}\left[\left(1-\psi_{t}\right)h_{t}^{h}n_{t}^{h}+\psi_{t}h_{t}^{l}n_{t}^{l}\right].$$ (3.14)

The individual pension benefits which have to be proportional to the income of the first period requires that the following holds:

$$P_{t+1}^{i}=\frac{h_{t}^{i}}{\left(\left(1-\psi_{t}\right)h_{t}^{h}+\psi_{t}h_{t}^{l}\right)}{\bar{P}}_{t+1}.$$ (3.15)

Inserting (3.15) in (3.11) delivers for the number of children:

$$n_{t}^{i}=\frac{\phi \left(1-\varepsilon \nu \right)\left(\left(1-\tau^{P}\right)R_{t}w_{t}+\frac{{\bar{P}}_{t+1}}{\left(\left(1-\psi \right)h_{t}^{h}+\psi h_{t}^{l}\right)}\right)}{R_{t}w_{t}e\left(1+\chi +\phi \right)}.$$ (3.16)

We notice that the number of children is no longer dependent on the ability of parents. Or in other words the number of children is equal for low and high ability individuals.

**Proposition 2**: In a Bismarckian system the number of children is independent of the ability of parents, thus $n_{t}^{h}=n_{t}^{l}=n_{t}$.

Using this outcome to calculate the individual pension benefit we get:

$$P_{t+1}^{i}=h_{t}^{i}\tau^{P}n_{t}G^{h}w_{t+1}.$$ (3.17)

Assuming a small open economy, in which the international capital market determines the interest factor and wage rate per human capital unit, we rewrite the wage rate per human capital unit as *w*_*t*_ = *w*_*t*+1_ = *w* and the interest factor as *R*_*t*_ = *R*_*t*+1_ = *R*.

we get after inserting (3.17) in Eqs (3.10) and (3.11), and solving for savings per capita and number of children:

$$s^{BIS*}=\frac{\left(1-\tau^{P}\right)\left(eR\chi -G^{h}\tau^{P}\left(1-\varepsilon \nu \right)\phi \right){\bar{h}}_{t}w_{t}}{eR\left(1+\chi +\phi \right)-\phi G^{h}\tau_{t}^{P}\left(1-\varepsilon \nu \right)}$$ (3.18)

$$n^{BIS*}=\frac{R\phi \left(1-\tau^{P}\right)\left(1-\varepsilon \nu \right)}{eR\left(1+\chi +\phi \right)-\phi G^{h}\tau_{t}^{P}\left(1-\varepsilon \nu \right)}$$ (3.19)

We can conclude that savings are proportional to individual human capital, while the number of children is independent of it. Consequently, the average per capita savings are proportional to the average human capital.

As for the analysis of the sustainability of the system, it must be noted that population shares of high and low-ability individuals remain unchanged. To address the long-run sustainability of the pension system, we need to consider the replacement ratio (Stauvermann and Kumar 2016)–the ratio between pension benefits and wage income, excluding contributions.


$$\omega_{t}=\frac{P_{t+1}^{i}}{\left(1-\tau^{P}\right)h_{t}^{i}w_{t}}=n^{BIS*}\;G^{h}=\frac{BR\phi \left(1-\tau^{P}\right){\left(1-\varepsilon \nu \right)}^{1-\varepsilon }{\left(\varepsilon \nu e\right)}^{\varepsilon }}{eR\left(1+\chi +\phi \right)-\phi G^{h}\tau_{t}^{P}\left(1-\varepsilon \nu \right)}>0,$$ (3.20)

Another important question to consider is how the ratio of consumption between the old and the young will evolve in a specific period. This question becomes particularly intriguing when addressing whether retirees can afford a relatively similar lifestyle to that of the workers. Like Stauvermann and Kumar (2016) we define $\Omega_{t}=\frac{c_{t+1}^{2}}{c_{t+1}^{1}}$

$$\Omega_{t}=\frac{\left(\left(eR\chi -G^{h}\tau^{P}\left(1-\varepsilon \nu \right)\phi \right)\right)\left(1-\tau^{P}\right)R+\tau^{P}BR\phi {\left(1-\varepsilon \nu \right)}^{1-\varepsilon }{\left(\varepsilon \nu e\right)}^{\varepsilon }}{G^{h}\left(eR\left(1+\chi +\phi \right)-\phi G^{h}\tau_{t}^{P}\left(1-\varepsilon \nu \right)-\left(1-\tau^{P}\right)\left(eR\chi -G^{h}\tau^{P}\left(1-\varepsilon \nu \right)\phi \right)\right)}>0.$$ (3.21)

Therefore, even if the population is growing at an unsustainable rate, the Bismarckian system is sustainable.

## 5. The Beveridgean pension scheme

And next, we derive the outcomes of the model in the presence of a Beveridgean pension system. In this system the pension benefits are equal for all retired individuals and a balanced budget requires in period *t*:

$$N_{t-1}P_{t}=\tau^{P}w_{t}\left(h_{t}^{l}\psi_{t}+h_{t}^{h}\left(1-\psi_{t}\right)\right)N_{t}$$ (3.22)

Under a purely Beveridgean pension scheme, the pension benefit is identical for all the workers:

$$P_{t+1}=\tau^{P}w_{t}\;\left(h_{t}^{l}\psi_{t}n_{t}^{l}+h_{t}^{h}\left(1-\psi_{t}\right)n_{t}^{h}\right)G^{h}.$$ (3.23)

Inserting (3.11) in (3.23) and then solving for the pension benefit delivers:

$$P_{t+1}=\frac{R\phi \left(1-\tau^{P}\right)G^{h}\tau^{P}\left(1-\varepsilon \nu \right)w_{t}\left(\left(1-\psi_{t}\right)h_{t}^{h}+\psi_{t}h_{t}^{l}\right)}{eR\left(1+\chi +\phi \right)-\phi G^{h}\tau_{t}^{P}\left(1-\epsilon \nu \right)}.$$ (3.24)

Since the average human capital stock per capita ${\bar{h}}_{t}=\left(1-\psi_{t}\right)h_{t}^{h}+\psi_{t}h_{t}^{l}$, we insert it in (3.24) and get:

$$P_{t+1}=\frac{R\phi \left(1-\tau^{P}\right)G^{h}\tau^{P}\left(1-\varepsilon \nu \right)w_{t}{\bar{h}}_{t}}{eR\left(1+\chi +\phi \right)-\phi G^{h}\tau_{t}^{P}\left(1-\varepsilon \nu \right)}$$ (3.25)

Now we can insert (3.25) in (3.10) and (3.11) to derive the optimal savings, and optimal number of children. We first get:

$$s_{t}^{l}=\frac{w_{t}h_{t}^{l}\left(1-\tau^{P}\right)\chi -\frac{\phi \left(1-\tau^{P}\right)G^{h}\tau^{P}\left(1-\varepsilon \nu \right)w_{t}{\bar{h}}_{t}\left(\phi +1\right)}{eR\left(1+\chi +\phi \right)-\phi G^{h}\tau_{t}^{P}\left(1-\varepsilon \nu \right)}}{\left(\phi +\chi +1\right)}$$ (3.26)

$$s_{t}^{h}=\frac{w_{t}h_{t}^{h}\left(1-\tau^{P}\right)\chi -\frac{\phi \left(1-\tau^{P}\right)G^{h}\tau^{P}\left(1-\varepsilon \nu \right)w_{t}{\bar{h}}_{t}\left(\phi +1\right)}{eR\left(1+\chi +\phi \right)-\phi G^{h}\tau_{t}^{P}\left(1-\varepsilon \nu \right)}}{\left(\phi +\chi +1\right)}$$ (3.27)

For the average savings, we take the weighted value of the low ability workers and the high ability workers, and get $\bar{s_{t}}=\;\psi s_{t}^{l}+\left(1-\psi \right)s_{t}^{h}$; we solve for this function and get

$$\bar{s_{t}}=\frac{\left(Re\chi -G^{h}\left(1-\varepsilon \nu \right)\tau^{P}\phi \right)\left(1-\tau^{P}\right){\bar{h}}_{t}w_{t}}{eR\left(1+\chi +\phi \right)-\phi G^{h}\tau^{P}\left(1-\varepsilon \nu \right)}$$ (3.28)

For fertility rates, we apply the same rule, and insert the pension benefit function into the fertility choice for the low ability workers and the high ability workers, and get

$$n_{t}^{l}=\frac{\phi \left(1-\tau^{P}\right)\left(1-\epsilon \nu \right)\left(Reh_{t}^{l}\left(\phi +\chi +1\right)+\phi G^{h}\left({\bar{h}}_{t}-h_{t}^{l}\right)\left(1-\epsilon \nu \right)\tau^{P}\right)}{e\left(\phi +\chi +1\right)h_{t}^{l}\left(Re\left(\phi +\chi +1\right)-\phi G^{h}\left(\epsilon \nu -1\right)\tau^{P}\right)}$$ (3.29)

$$n_{t}^{h}=\frac{\phi \left(1-\tau^{P}\right)\left(1-\varepsilon \nu \right)\left(Reh_{t}^{h}\left(\phi +\chi +1\right)+\phi G^{h}\left({\bar{h}}_{t}-h_{t}^{h}\right)\left(1-\varepsilon \nu \right)\tau^{P}\right)}{e\left(\phi +\chi +1\right)h_{t}^{h}\left(Re\left(\phi +\chi +1\right)-\phi G^{h}\left(\varepsilon \nu -1\right)\tau^{P}\right)}$$ (3.30)

The average number of children is ${\bar{n}}_{t}=\;\psi_{t}n_{t}^{l}+\left(1-\psi_{t}\right)n_{t}^{h}$; we solve for this function and get

$${\bar{n}}_{t}=\frac{\left(\left(1-\varepsilon \nu \right)G^{h}\left(\frac{\left(1-\psi_{t}\right){\bar{h}}_{t}}{h_{t}^{h}}+\frac{{\bar{h}}_{t}\psi_{t}}{h_{t}^{l}}-1\right)\tau^{P}\phi +Re\left(\chi +1+\phi \right)\right)\left(1-\varepsilon \nu \right)\left(1-\tau^{P}\right)\phi }{e\left(\phi +\chi +1\right)\left(Re\left(\phi +\chi +1\right)-\phi G^{h}\left(1-\varepsilon \nu \right)\tau^{P}\right)}$$ (3.31)

**Proposition 3**: In this model the application of a Beveridgean pension system leads to the outcome that fertility of the low ability workers exceeds the fertility rate of the high ability workers.

Proof: Taking the fertility rate of the low ability workers (3.29) and the fertility rate of the high ability workers (3.30) and assuming that $n_{t}^{l}>n_{t}^{h}$, we get after a few reformulation:

$$\frac{\left({\bar{h}}_{t}-h_{t}^{l}\right)}{h_{t}^{l}}>\frac{\left({\bar{h}}_{t}-h_{t}^{h}\right)}{h_{t}^{h}}.$$

Because of the fact that $h_{t}^{h}>{\bar{h}}_{t}>h_{t}^{l}$ holds by definition, the RHS of the inequality above is always negative and the LHS always positive. Q.E.D.

However, if the fertility rates of both groups differ, the distribution of low and high ability workers is affected.

**Proposition 4**: In our model, the application of a Beveridgean pension system will lead to a decrease of the high ability workers.

**Proof**: The share of low ability workers in period t is defined as $\psi_{t}=\frac{N_{t}^{l}}{N_{t}}$ and accordingly the share of low ability workers in period t+1 is then given by $\psi_{t+1}=\frac{n_{t}^{l}N_{t}^{l}}{{\bar{n}}_{t}N_{t}}$. The growth factor of the share of low ability workers becomes:

$$\frac{\psi_{t+1}}{\psi_{t}}=\frac{n_{t}^{l}}{{\bar{n}}_{t}}>1,\;\forall t.$$

From this result, we can further derive the paradoxical outcome:

**Proposition**: Despite that the human capital in each group of the economy grows with the factor *G*^*h*^ the average human capital grows at a lower speed.

Proof: The average human capital in period t is defined by:

$${\bar{h}}_{t}=\psi_{t}h_{t}^{l}+\left(1-\psi_{t}\right)h_{t}^{h}$$

and the average human capital in period t+1 is given by:

$${\bar{h}}_{t+1}=\frac{\left(\psi_{t}h_{t}^{l}n_{t}^{l}+\left(1-\psi_{t}\right)h_{t}^{h}n_{t}^{h}\right)G^{h}}{{\bar{n}}_{t}}.$$

Then, the growth factor of the average human capital $G_{\bar{h}}$ becomes to

$$G_{\bar{h}}=\frac{\left(\psi_{t}h_{t}^{l}\frac{n_{t}^{l}}{{\bar{n}}_{t}}+\left(1-\psi_{t}\right)h_{t}^{h}\frac{n_{t}^{h}}{{\bar{n}}_{t}}\right)G^{h}}{\left(\psi_{t}h_{t}^{l}+\left(1-\psi_{t}\right)h_{t}^{h}\right)}=\frac{\left(\psi_{t+1}h_{t}^{l}+\left(1-\psi_{t+1}\right)h_{t}^{h}\right)G^{h}}{\left(\psi_{t}h_{t}^{l}+\left(1-\psi_{t}\right)h_{t}^{h}\right)}<G^{h}.$$

It is straightforward to show that $\frac{\left(\psi_{t+1}h_{t}^{l}+\left(1-\psi_{t+1}\right)h_{t}^{h}\right)}{\left(\psi_{t}h_{t}^{l}+\left(1-\psi_{t}\right)h_{t}^{h}\right)}<1$. This result also implies that the outcome of Hachon [29] does only hold in a world with positive externalities of capital and exogenous population growth.

## 6. Welfare considerations and discussion

As demonstrated above, the replacement ratio of a PAYG pension system tends to stabilize at a fixed value, contingent on preference parameters and the contribution rate of the pension system. Stauvermann and Kumar [1] have shown that this ratio remains unaffected even if we take into account increasing life expectancy. In the long run, the replacement rate is identical for both types of PAYG pension systems. The main distinction between these systems lies in the fact that, in an economy with a Bismarckian pension system, the average income and pension benefit are higher, but the low-ability individuals are not better off compared to those in an economy with a Beveridgean pension system.

When considering potential preferences regarding status or absolute consumption levels, it becomes evident that the welfare in an economy with a Beveridgean pension system will increase despite declining pension benefits if individuals are primarily concerned with relative utility. This is because the Beveridgean system reduces inequality in the long run. However, if only relative utility matters, societal welfare is maximized when all members receive the same income and have equal opportunities. On the other hand, if individuals are solely interested in the absolute level of consumption, the Beveridgean pension system is inferior compared to the Bismarckian one.

The outcome is probably not surprising; hence, the more important question is whether the absolute or relative level of consumption is more relevant for individuals. There are several reasons that indicate the importance of the relative consumption theory. In summary, the following reasons can be provided:

Firstly, the social comparison theory, introduced by Festinger (1954) [30], states that human beings tend to evaluate their well-being and happiness based on comparisons with others in their social circle. In developed countries, where access to basic necessities is widespread, individuals are more likely to gauge their satisfaction and welfare by assessing their relative standing compared to their peers, rather than solely focusing on absolute material possessions. While the absolute level of consumption is undeniably influential for well-being when basic necessities are not accessible, its importance diminishes as necessities become more attainable.

Secondly, developed countries often exhibit significant income inequality. As the gap between the wealthy and the less affluent widens, relative consumption becomes increasingly crucial for individuals. Those with lower incomes may feel deprived and experience reduced welfare when they observe conspicuous consumption by the affluent, even if their absolute consumption levels are objectively sufficient. For instance, the absolute level of consumption of a lower middle-class citizen in wealthy EU countries undoubtedly surpasses the consumption level of kings and queens from past centuries, considering factors such as healthcare access, media, electricity, heating, air conditioning, cars, and airplanes. However, it is challenging to argue that lower middle-class citizens achieve a higher level of life satisfaction as a result.

Thirdly, social cohesion is an important factor for individual well-being and happiness, as shown by Delhey and Dragolov (2016) [31]. Relative consumption significantly influences social cohesion and happiness levels in developed societies. Large disparities in wealth and consumption can lead to social divisions and erode sense of community, negatively impacting overall well-being. On the other hand, when relative consumption is more balanced, it fosters a sense of unity and shared prosperity, contributing to higher levels of welfare and life satisfaction.

The concept of hedonic adaptation, or hedonic treadmill, suggests that as people’s absolute wealth increases, they tend to adapt to their new material circumstances, leading to diminishing returns in terms of happiness (Brickman and Campbell 1971) [32]. However, relative consumption comparisons can continue to influence well-being, as people’s desire for social status and recognition remains ongoing. This concept of hedonic adaptation contradicts the notion that the absolute consumption level is solely relevant for individual well-being.

Another argument can be based on psychological and societal norms, as societal norms and expectations often revolve around relative status and material success. People may be driven by the pursuit of relative superiority, recognition, and respect, which can have a more significant impact on their overall welfare than the mere accumulation of material goods. Clear examples of such relative status races include the increasing use of rankings measuring the relative success of a person, institution, or company.

In societies with high income inequality, the perception of relative deprivation can lead to chronic stress, and are associated with adverse health effects, impacting individual well-being.

In conclusion, relative consumption appears to be more relevant for the welfare of people in developed countries than the absolute level of consumption. Addressing relative disparities and fostering a sense of social cohesion and fairness can contribute to higher overall well-being and life satisfaction in these societies.

## 7. Conclusions

In this paper, we delve into the long- and short-run consequences of the Bismarckian and Beveridgean pension systems. Our findings reveal that the Bismarckian system outperforms the Beveridgean system in terms of per capita GDP due to the constancy of the distribution of high and low-ability workers in countries employing it, whereas the number of high-ability workers decreases in an economy with a Beveridgean system.

The reason behind this lies in the redistributive element present in the Beveridgean system, which leads to an increase in the fertility of low-ability workers and a decrease in the fertility rate of high-ability workers. The underlying mechanism applies to both groups but with opposite effects. High-ability workers, receiving relatively low pension benefits, compensate for this by increasing their savings, resulting in fewer children. On the other hand, low-ability workers, benefiting from relatively higher pension benefits, reduce their savings and invest more in the number of children.

Furthermore, our analysis demonstrates that both PAYG pension systems are sustainable, as the replacement ratio consistently remains above zero. When considering different preferences, the choice of pension system should depend on whether individuals prioritize the absolute level of consumption or consumption relative to a reference group. If the latter is more crucial, the Beveridgean system proves superior, providing greater protection for vulnerable groups than the Bismarckian pension system. In this case, the pension income after retirement is relatively more balanced, but the trade-off is a lower level of consumption in the long run compared to an economy with a Bismarckian system. Conversely, if individuals prioritize the absolute level of consumption, the Bismarckian system performs better, ensuring a comparatively higher level of consumption. However, it comes with the drawback of higher poverty risks for disadvantaged groups and relatively greater social inequality.

It is essential to note that in the long run, only the level of consumption differs between the two systems, not the speed of growth or the number of children.
